# Sustained Hematological Stability During Clozapine Rechallenge With Adjunctive Lithium: A Case Report of 15-Month Follow-Up

**DOI:** 10.7759/cureus.108986

**Published:** 2026-05-16

**Authors:** Karim Hamda, Azeddine Barkiat, Siham Belbachir, Abderrazzak Ouanass

**Affiliations:** 1 Department of Psychiatry, Arrazi Hospital, Ibn Sina University Hospital Center, Faculty of Medicine and Pharmacy, Mohammed V University, Rabat, MAR

**Keywords:** case report, clozapine, lithium, neutropenia, treatment-resistant schizophrenia

## Abstract

Clozapine is the most effective treatment for treatment-resistant schizophrenia, offering significant therapeutic benefits for patients who have failed to respond to other antipsychotic regimens. However, its clinical use is limited by the risk of severe hematological adverse events, particularly neutropenia and agranulocytosis. Lithium has been reported to induce leukocytosis through the stimulation of granulopoiesis and has been considered as a potential adjunctive strategy in the management of clozapine-associated neutropenia. However, long-term hematological outcomes of combined clozapine-lithium therapy remain insufficiently documented, particularly at subtherapeutic lithium levels.

We report the case of a 32-year-old man with treatment-resistant schizophrenia and a prior history of clozapine-induced neutropenia. Following multiple antipsychotic treatment failures, clozapine was reintroduced and combined with low-dose adjunctive lithium (400 mg/day). Hematological and biochemical monitoring was conducted over a 15-month period, during which 25 sequential complete blood counts were analyzed.

The mean leukocyte count was 9.23 ± 1.68 × 10³/mm³ (range: 5.55-13.07 × 10³/mm³), and the mean neutrophil count was 5.81 ± 1.49 × 10³/mm³ (range: 2.53-8.86 × 10³/mm³). No episodes of neutropenia were observed during follow-up. A strong positive correlation was found between total leukocyte and absolute neutrophil counts (r = 0.97; p < 0.001). Linear regression analysis showed no significant temporal decline in leukocyte levels (p = 0.846). Lithium plasma levels remained between 0.17 and 0.54 mmol/L, below the standard therapeutic range for mood stabilization. The patient showed clinical improvement and maintained global functioning without further psychiatric hospitalizations.

This case suggests a possible role of adjunctive lithium in supporting hematological stability during clozapine therapy. Subtherapeutic lithium levels may be associated with stable leukocyte and neutrophil counts in selected cases; however, no causal relationship can be established. This approach should be considered exploratory and requires strict hematological monitoring.

## Introduction

Clozapine is widely regarded as the gold standard for the management of treatment-resistant schizophrenia, offering significant therapeutic benefits for patients who have failed to respond to other antipsychotic regimens [1]. However, its clinical use is limited by the risk of hematological adverse events, particularly neutropenia and agranulocytosis [2,3]. Neutropenia occurs in approximately 2-3% of patients treated with clozapine, while agranulocytosis is rarer (0.5-1%) but remains potentially life-threatening [2,3]. These complications represent a major limitation of clozapine therapy and necessitate regular monitoring of the absolute neutrophil count (ANC). In some cases, they may lead to treatment discontinuation, exposing patients to a high risk of relapse [4,5].

Lithium, a well-established mood stabilizer, is known to induce leukocytosis through the stimulation of granulopoiesis. This effect has led to its use as a potential adjunctive strategy in cases of clozapine-associated neutropenia, particularly to support treatment continuation or rechallenge [6]. However, the available evidence remains limited and is primarily derived from case reports and small observational studies. In addition, long-term data on the hematological stability of this combination are scarce, especially when lithium is used at subtherapeutic plasma levels to minimize its own adverse effects.

In this context, we report a 15-month longitudinal follow-up of a 32-year-old man with treatment-resistant schizophrenia and a history of clozapine-induced neutropenia. This report aims to describe the clinical and hematological course during clozapine rechallenge with adjunctive lithium and to explore the potential role of low-dose lithium in maintaining hematological stability.

## Case presentation

A 32-year-old man with a 10-year history of treatment-resistant schizophrenia presented with persistent psychotic symptoms despite multiple antipsychotic trials, including risperidone and olanzapine.

A previous trial of clozapine, administered at a dose of 400 mg/day, was discontinued in 2019 following the development of neutropenia (ANC <1,500/mm³). No alternative causes, such as infection or concomitant medications, were identified. Neutrophil counts returned to normal within a few weeks after clozapine discontinuation.

Following clozapine withdrawal, the patient received several alternative antipsychotic treatments; however, none resulted in adequate or sustained clinical improvement. By 2024, the patient remained clinically unstable, with persistent symptoms and impaired functioning.

Given the persistence of symptoms and the lack of effective therapeutic alternatives, a clozapine rechallenge was initiated in 2024 in combination with adjunctive lithium at a dose of 400 mg/day.

**Figure 1:**
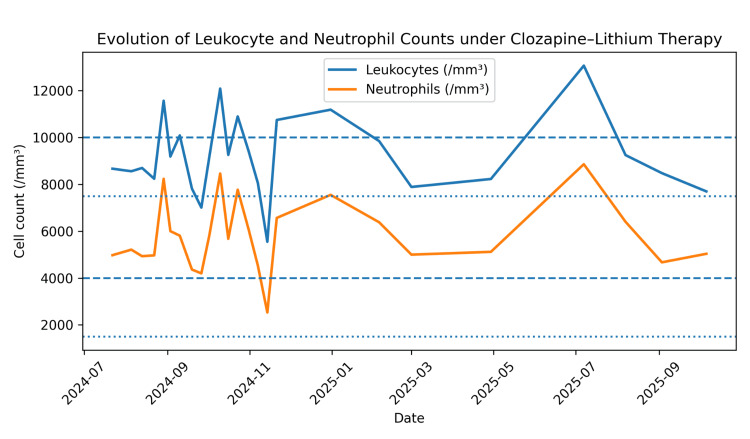
Longitudinal changes in leukocyte and neutrophil counts during clozapine-lithium therapy Evolution of leukocyte and neutrophil counts during clozapine-lithium therapy over a 15-month follow-up period. Leukocyte and neutrophil levels remained stable without episodes of neutropenia.

Over a 15-month follow-up period, 25 sequential complete blood counts were performed. The mean leukocyte count was 9.23 ± 1.68 × 10³/mm³, and the mean neutrophil count was 5.81 ± 1.49 × 10³/mm³. No episodes of neutropenia (ANC <1,500/mm³) were recorded during the monitoring period. A strong positive correlation was found between total leukocyte and ANC (r = 0.97; p < 0.001) (Figure 1). Linear regression analysis showed no significant temporal decline in leukocyte counts (p = 0.846).

Lithium plasma levels were regularly monitored and ranged between 0.17 and 0.54 mmol/L, remaining below the conventional therapeutic range for mood stabilization. Following clozapine reintroduction, the patient showed clinical improvement, including better adherence to follow-up appointments, reduction in psychotic symptoms, and improved behavioral stability, although mild daytime sedation was reported during treatment.

## Discussion

The management of clozapine-induced neutropenia remains a significant challenge in clinical practice. This case adds to the existing literature suggesting a potential role for adjunctive lithium in supporting hematological stability during clozapine rechallenge [1,2,7]. The most notable observation in this 15-month follow-up is the maintenance of stable leukocyte and neutrophil counts despite lithium plasma levels remaining below the conventional therapeutic range for mood disorders.

A strong positive correlation was found between leukocyte and neutrophil counts (r = 0.97; p < 0.001) (Figure 1). While this finding is consistent with the known granulopoietic effects of lithium, it should be interpreted with caution, as causality cannot be established in a single-case observation. Similarly, the absence of a significant temporal decline in leukocyte levels may suggest ongoing hematological stability, although natural variability cannot be excluded.

Lithium-induced leukocytosis is thought to involve the stimulation of granulopoiesis through the increased production of colony-stimulating factors and enhanced bone marrow activity. In this case, hematological stability was observed despite subtherapeutic lithium levels, raising the hypothesis that low-dose lithium may be associated with such effects. However, this observation remains exploratory and cannot be generalized.

Importantly, this approach should not be considered a standardized or predictive strategy for preventing clozapine-associated neutropenia. Neutropenia may still occur despite lithium use, and strict hematological monitoring remains essential in all cases.

This report has several limitations. It describes a single patient, limiting generalizability, and the absence of a control condition precludes causal inference. Therefore, these findings should be considered hypothesis-generating and require confirmation in larger, prospective studies.

## Conclusions

This 15-month longitudinal observation suggests that adjunctive lithium may be associated with hematological stability during clozapine rechallenge in patients with a history of neutropenia. These findings should be interpreted with caution given the single-case design, and no causal relationship can be established.

In this context, subtherapeutic lithium exposure was observed alongside stable leukocyte and neutrophil counts, raising the hypothesis that low-dose lithium may contribute to such effects in selected cases. However, this approach remains exploratory and requires strict hematological monitoring.

Further studies are needed to confirm these observations and to better define the role, optimal dosing, and safety of lithium as an adjunctive strategy during clozapine rechallenge.
